# Profiling and Validation of the Circular RNA Repertoire in Adult Murine Hearts

**DOI:** 10.1016/j.gpb.2016.02.003

**Published:** 2016-04-27

**Authors:** Tobias Jakobi, Lisa F. Czaja-Hasse, Richard Reinhardt, Christoph Dieterich

**Affiliations:** 1 Section of Bioinformatics and Systems Cardiology, Department of Internal Medicine III, University Hospital Heidelberg, 69120 Heidelberg, Germany; 2 German Centre for Cardiovascular Research (DZHK), Heidelberg/Mannheim, Germany; 3 Max Planck-Genome-Centre Cologne, 50829 Cologne, Germany

**Keywords:** Circular RNA, circSeq, Cardiovascular disease, Cardiomyopathy, Computational cardiology

## Abstract

For several decades, **cardiovascular disease** has been the leading cause of death throughout all countries. There is a strong genetic component to many disease subtypes (*e.g.*, **cardiomyopathy**) and we are just beginning to understand the relevant genetic factors. Several studies have related RNA splicing to **cardiovascular disease** and **circular RNAs** (circRNAs) are an emerging player. circRNAs, which originate through back-splicing events from primary transcripts, are resistant to exonucleases and typically not polyadenylated. Initial functional studies show clear phenotypic outcomes for selected circRNAs. We provide, for the first time, a comprehensive catalogue of RNase R-resistant circRNA species for the adult murine heart. This work combines state-of-the-art circle sequencing with our novel DCC software to explore the circRNA landscape of heart tissue. Overall, we identified 575 circRNA species that pass a beta-binomial test for enrichment (false discovery rate of 1%) in the exonuclease-treated sequencing sample. Several circRNAs can be directly attributed to host genes that have been previously described as associated with *cardiovascular disease*. Further studies of these candidate circRNAs may reveal disease-relevant properties or functions of specific circRNAs.

## Introduction

Circular RNAs (circRNAs) are a recently-rediscovered RNA class, which were initially described as “scrambled” exons in the early 1990s [1]. Those structures of out-of-order exons may either result from tandem exon duplications, *trans*-splicing, or joining splice donor and acceptor sites of a transcript, thus creating a circular construct. In 2013, several ground-breaking manuscripts demonstrated a clear phenotypic outcome after circRNA perturbation and sparked enormous interest in this molecular species [2], [3], [4]. Since then, a plethora of manuscripts on circRNA predictions in various contexts has appeared in the literature. circRNAs are typically identified in rRNA-depleted sequencing samples as back-splicing events [5] that break the collinearity between RNA and DNA sequences. However, this necessary condition is not sufficient to prove circularity of the RNA species, since “scrambled exons” may also arise from *trans*-splicing and tandem duplications as mentioned above, genomic alterations, or simply from read mapping artefacts [6].

The identification and quantification of circRNAs from next-generation sequencing (NGS) data is an area of ongoing research. The “circSeq” approach is the current gold standard for high-throughput circRNA identification [7]. Briefly, circRNAs are resistant to exonuclease treatment, whereas linear RNAs are selectively depleted with this approach. An untreated control sample and an RNase R-treated sample are subjected to RNA-seq and contrasted with one another. Although circSeq is the current gold standard, it may not work equally well on all circRNAs. For instance, CDR1as/ciRS-7 could not be enriched with RNase R treatment [8].

Relative abundance changes in circRNA expression are typically approximated by comparing normalized back-splice junction read counts of specific circRNAs under different conditions and/or time points [9]. Moreover, we recently presented a statistical framework to detect host gene-independent circRNA expression changes from sequencing data [10].

It is widely assumed that circRNAs belong to the growing group of non-coding RNAs (ncRNAs) that encompasses microRNAs (miRNAs), long ncRNAs (lncRNA), or small-interfering RNAs (siRNA). Intriguingly, a recent study in endothelial cells showed that circRNAs are regulated in response to hypoxia and have a biological function as proven by *in vitro* experiments [11]. Nonetheless, no rigorous reports have been published on circRNAs in murine or human heart cell types. An extrapolation from other tissue or cell type data is not possible as previous analyses have clearly demonstrated that a large proportion of circRNAs are cell type specific [12].

In this manuscript, we aim to provide a catalogue of validated circRNA species in the adult murine heart. To this end, we employ the latest developments in bioinformatics and sequencing technology, and relate our catalogue to previously-identified cardiomyopathy-related genetic loci.

## Results and discussion

Total RNA was extracted from hearts of adult mice which were 2, 3, 6, or 12 months old. After depletion of rRNA, each RNA sample was split into two fractions with one treated with RNase R (RNase R^+^) and the other left untreated (RNase R^−^) (see Methods). No polyA-containing RNA removal step was carried out, in accordance with other recent reports on fragment-based circRNA detection [7] and due to the fact that otherwise no comparison of linear transcript counts to circular counts would be possible. We prepared cDNA libraries from these 8 samples and sequenced them on an Illumina HiSeq 2500. The corresponding read coverage tracks have been compiled into a publicly-available TrackHub for the UCSC Genome Browser [13] (Supplementary online data).

### 575 circRNAs are significantly enriched

The DCC software [10] was used to identify circRNA candidates from chimeric read mappings (see Materials and Methods). This initial step yielded 8375 potential circRNA regions. We subsequently employed the *circTest* suite in DCC [10] to confine the search to 741 circRNA loci by filtering for consistent read support and a minimal proportion of junction-spanning reads (1%). Lastly, we tested enrichment of back-splicing in read sets of RNase R^+^ in comparison to RNase R^−^ samples and further reduced the candidate circRNA set to 575 circRNAs (Table S1). We additionally screened the catalogue of the 575 significant circRNAs for alternative splicing using the DCC output files and found only 2.4% having >3 supporting reads for alternative splicing variants.

**Figure 1:**
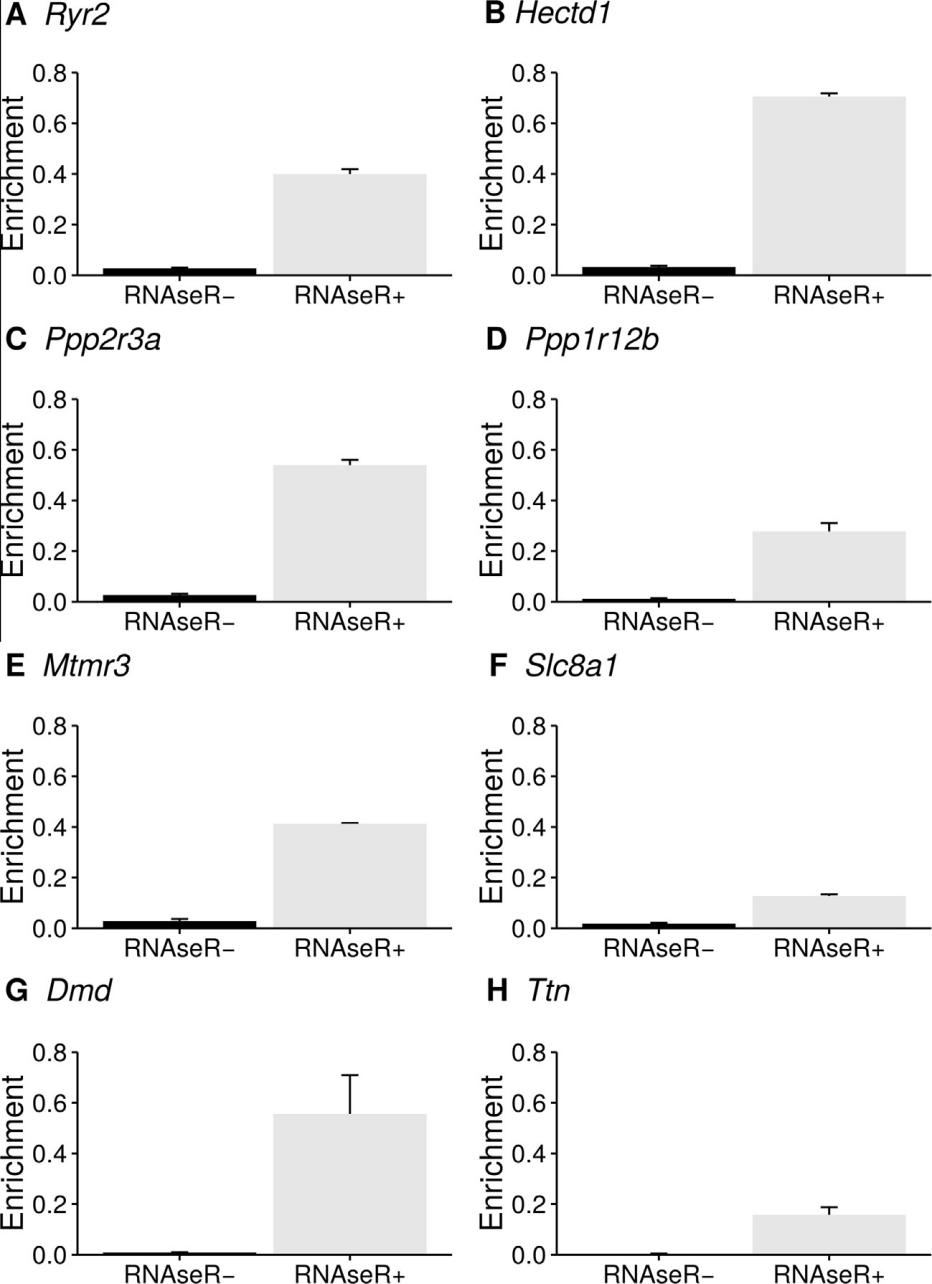
Comparison of the enrichment for circRNAs in RNase R^+^ and RNase R^−^ samples The 8 most significant circRNAs assessed by *circTest* and linked to certain types of cardiovascular disease are shown (BH corrected *P* values, FDR 1%). *circTest* uses the beta binomial distribution to model the data and performs an ANOVA to identify circRNAs that differ in their relative expression between the groups. Due to the implementation of *circTest*, the 4 time points for the same samples are treated as 4 replicates (*n* = 4) for RNase R treated (RNase R^+^) and untreated (RNase R^−^) samples. Error bars show the standard error of the mean. Therefore, changes in expression at all 4 time points are taken into account when calculating the host gene independence. *Ryr2,* ryanodine receptor 2; *Hectd1*, HECT domain-containing E3 ubiquitin protein ligase 1; *Ppp2r3a*, protein phosphatase 2, regulatory subunit B; Ppp1r12b, protein phosphatase 1, regulatory subunit 12B; *MTMR3*, myotubularin-related protein 3; *Slc8a1*, solute carrier family 8 (sodium/calcium exchanger), member 1; *Dmd*, dystrophin; Ttn, titin.

Table 1 shows an example of 8 selected circRNAs that are localized in cardiovascular disease-related gene loci. *circTest* generates enrichment plots for each of the significant circRNAs (BH corrected *P* values, FDR 1%). Due to the employed circle sequencing approach, RNase R^−^ and RNase R^+^ samples should be clearly separated within the enrichment plot since the RNase R digestion step removes the bulk of linear transcripts and more sequencing reads actually cover circRNAs structures instead of linear transcripts in the untreated sample. Therefore, this graphical representation is a first quality measurement for the success of enrichment process. In an ideal experiment all linear fragments in the RNase R treated sample should be digested, leaving only circular constructs in the sample. A drastic increase of reads covering circRNAs would therefore be expected when comparing untreated and treated samples. Also this ideal situation does not translate exactly to practice. The enrichment plots in Figure 1 show that the enrichment process performed well for all samples, thus confirming the gold standard of circle sequencing.

### Inspection of the most significant circRNAs

The vast majority of circRNAs were only found at only one location within the annotated gene regions (97.6%, threshold: 3 supporting reads). For the remaining 2.4% circRNAs, more than 3 reads were found at a secondary location within the annotated gene regions; however, hardly more than 10 reads were detected at the secondary locations for these circRNAs. Therefore, circRNAs seem to be predominantly emerging from single, specific gene regions. Among the 575 significant circRNAs identified above, we selected the top three most significant loci, *Ryr2*, *Hectd1*, and *Ppp2r3a* that encode ryanodine receptor 2, HECT domain-containing E3 ubiquitin protein ligase 1, and protein phosphatase 2, regulatory subunit B, respectively, for further inspection.

**Figure 2:**
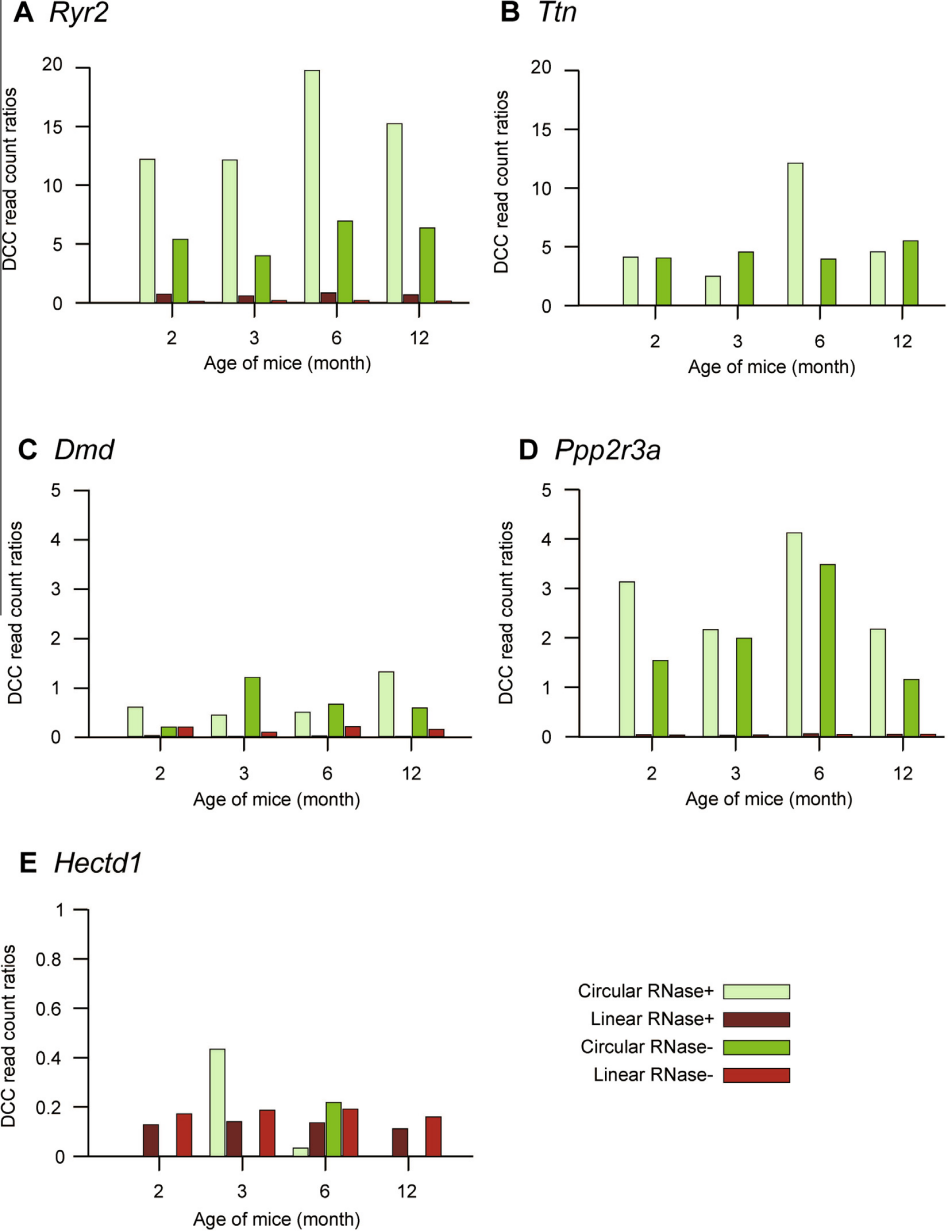
Detailed view of five selected genes strongly related to cardiovascular disease The ratio is calculated as (raw count of mapped reads for transcript in sample/raw count of total reads in sample) * 1000) for *Ryr2* (**A**), *Ttn* (**B**), *Dmd* (**C**), *Ppp2r3a* (**D**), and *Hectd1* (**E**).

**Figure 3:**
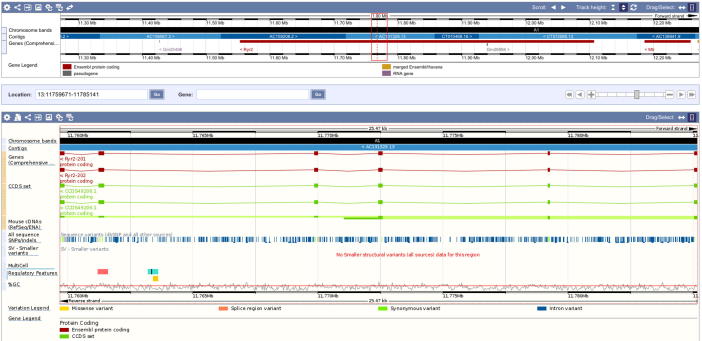
Ensembl screen shot of the circRNA locus linked to the *Ryr2* gene The region covered by the circRNA locus is defined by the red rectangle, which measures around 25 kb and covers 6 exons of *Ryr2*. Mouse genome is shown as black/white line and tracks depict the exon/intron structure of the gene.

The annotated genomic region of *Ryr2*, throughout all experiments, showed the most significant enrichment in general (Table 1, Figure 1). The combination of expression level, enrichment compared to the remaining linear RNAs reported by *circTest* (Figure 2), and its involvement in arrhythmias make *Ryr2* circRNA a very promising candidate for further analyses. The circRNA for *Ryr2* is roughly located between the second and last third of the annotated gene region (Figure 3). The RNase R^−^ circRNA candidate reads cover a region of 6 exons and 5 introns, starting at exon 24 and up to exon 29 of 25 kb in length.

**Figure 4:**
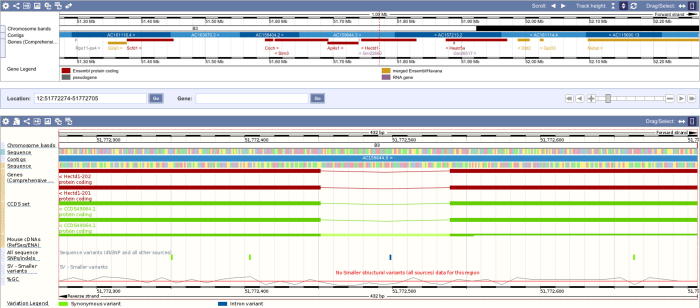
Ensembl screen shot of the circRNA locus linked to the *Hectd1* gene The region covered by the circRNA locus is defined by the red dotted line. Mouse genome is shown as black/white line below and tracks depict the exon/intron structure of the gene.

The locus of the *Hectd1* circRNA spans only 432 bp. The fragment spans only 2 exons (namely exon 23 and exon 24) and is located within the last third of *Hectd1* coding region (Figure 4). *Hectd1* circRNA belongs to the class of relatively small circRNAs. Notwithstanding, it is one of the most significant circRNAs in our samples.

**Figure 5:**
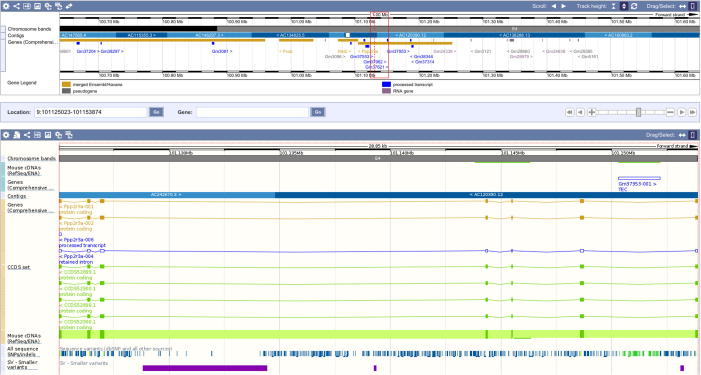
Ensembl screen shot of the circular RNA locus linked to the *Ppp2r3a* gene The region covered by the circRNA locus is defined by the red rectangle, which measures around 25 kb and covers 6 exons of *Ppp2r3a*. Mouse genome is shown as black/white line below and tracks depict the exon/intron structure of the gene.

In contrast, the identified circRNA locus of *Ppp2r3a* again measures more than 28 kb and contains 6 exons, starting from exon 6 and ranging up to exon 12. Due to the small size of *Ppp2r3a*, the 28 kb circRNA makes up nearly a quarter of the gene’s length and is located in the last quarter of the annotated gene region (Figure 5).

### Identification of candidate cardiovascular circRNAs

circRNAs are known to be highly specific to tissue and developmental status of the cell [12]. An in-depth analysis of highly-abundant circRNAs disclosed several genes tightly linked to functions of cardiomyocytes. Due to the selection of heart tissue and our focus on cardiovascular disease, we focused on three genes tightly coupled to cardiomyocytes and cardiomyopathy, *Ryr2*, *Ttn*, and *Dmd* for scrutinization.

These candidate genes are known to play key roles in different kinds of cardiomyopathy such as *Dmd*-associated dilated cardiomyopathy, arrhythmogenic right ventricular cardiomyopathy (ARVC) (*Ryr2*), or hypertrophic cardiomyopathy (HCM, *Ttn*), and therefore are central parts of the respective KEGG pathways (mmu05414, mmu05410, and mmu05412) [22].

There is an overwhelming presence of *Ryr2* circRNAs regardless of the age of the murine heart examined (Figure 2A). Primarily expressed in cardiomyocytes, *Ryr2* encodes ryanodine receptor 2 (RYR2), which is a sarcoplasmic reticulum membrane-embedded transport protein for Ca^2+^ and is responsible for the rhythmic muscle contractions generating the heartbeat [14]. The abundance of *Ryr2* circRNAs is relatively constant for all 4 time points, which peaks at month 6. Traces of *Ryr2* circRNA are detectable even without the enrichment step, whereas after enrichment the abundance is 2–3 times higher when compared to the untreated sample.

The detection of *Ttn* fits in the same picture, as it is responsible for the passive elasticity of muscles and therefore plays a critical role in heart muscle cells [21]. circRNA abundance of *Ttn* generally follows the pattern of *Ryr2* albeit with relatively modest changes. In comparison, the enrichment process was not as successful as for *Ryr2*, as higher circRNA counts were detected for month 4 and month 6 in the untreated samples than the treated samples. For example, very long circRNA molecules may get nicked or sheared during the isolation process, making them sensitive to RNAse R treatment.

*Dmd*-associated dilated cardiomyopathy is directly linked to mutations in the *Dmd* gene [20]. Therefore, *Dmd* circRNA could be another valuable target for cardiomyopathy research. The expression pattern of *Dmd* circRNA differs from those of *Ryr2* and *Ttn*, demonstrating maximal expression for month 12 instead of month 6. We achieved non-optimal enrichment for circRNAs from mice aged 3 or 6 months, possibly due to portions of the circRNAs lost during the RNase R digestion process.

The pattern of abundance of circRNAs for all three genes may be influenced by the age and activity of the mice. While the linear transcripts *Ryr2* and *Ttn* may be more connected to young and active hearts, *Dmd*-encoded protein strengthens the cardiomyocyte sarcolemmal integrity, and its maximal expression may be important for optimal function (hemodynamic and stress responses) of the fully developed, mature adult heart. Although the described properties are related to the protein-coding linear transcripts, their corresponding circRNAs may be involved in those diseases directly or indirectly.

## Conclusion

In this work we present circle sequencing as a highly efficient and sensitive tool for circRNA studies in cardiology. In summary, we compiled a catalogue of 575 candidate circRNAs that pass a test for enrichment in RNase R-treated samples in comparison to untreated samples using *circTest*. Many of these candidates coincide with disease-associated gene loci. For instance, significant candidates originate from the *Ryr2*, *Hectd1*, and *Ppp2r3a* gene loci that have been linked to cardiovascular disease.

As a long term goal, how these circRNAs might influence cell processes in disease-related phenotypes and healthy tissue needs to be addressed. Furthermore, more efforts are also needed as to how this information may translate into new treatment approaches of cardiovascular disease.

## Materials and methods

### Circle sequencing

Total RNA extracted from heart tissue of CD1-strain mice of ages 2, 3, 6, and 12 months was commercially acquired from AMS Biotechnology (Abingdon, Oxfordshire, UK). The rRNA depletion step was carried out with 20 μg of total RNA using the Ribo-Zero Magnetic Kit (Human, Mouse, Rat; Epicentre, Madison, WI, United States) and 5 μg input per reaction. Depletion was performed in parallel, followed by subsequent pooling. For digestion of linear RNA, 3 quarters of the depleted RNA was treated with RNase R, whereas the remaining one quarter of the depleted RNA only received water and acts as a negative control. Both samples were treated equally for all further steps. After heating to 70 °C and cooling to 35 °C, 10× RNase R reaction buffer and RNase R (Epicentre; Cat No. RNR07250) were added to the 3-quarter fraction, followed by heating to 37 °C for 40 min. Samples were cleaned up using Agencourt RNAClean XP beads (Beckman Coulter GmbH, Krefeld, North Rhine-Westphalia, Germany). Subsequent library preparation was performed with ScriptSeq-v2 RNA-Seq Library Preparation Kit (Epicentre). PCR amplification was performed with 15 cycles, without any further size selection. Sequencing was carried out on an Illumina HiSeq2500 system for 100 cycles using paired-end mode. Sequence data were deposited in the NCBI Sequence Read Archive (SRA) with the accession number SRP071584.

### Bioinformatics analyses

Paired-end rRNA-depleted sequencing data of RNase R treated and untreated samples were analysed in detail. After initial quality assessment, low quality regions and adapter sequences were removed with Flexbar (version 2.5) [23]. Read mapping against the mouse reference genome build mm10 was performed with the STAR aligning software (version 2.4.2a) [24].

To optimally exploit the advantage of the circSeq technique, we have developed the DCC software to efficiently detect and quantify circRNAs in sequencing data [10]. DCC software computes expression levels of host genes and circRNAs and allows for tests of circRNA expression independent of linear host genes even across several experimental conditions and replicates. DCC analysis took place with version 0.3.2 using the parameters “*-M -Nr 2 2 -fg -G -F -L 20*”. Further statistical analyses were performed with the *circTest* R package provided with DCC and additional custom-tailored Perl scripts.

## Authors’ contributions

CD designed the study; LC and RR performed all RNA and sequencing experiments. TJ and CD analysed the data and wrote the manuscript. All authors read and approved the final manuscript.

## Competing interests

The authors declared that there are no competing interests.

## Figures and Tables

**Table 1:** Selection of 8 significant circRNAs linked to certain types of cardiovascular disease

**Chr**	**Start**	**End**	**Gene**	**Locus**	**Ensembl ID**	**BH corrected *P* value**	**Linked disease**	**Ref.**
13	11759671	11785141	*Ryr2*	GRCm38:CM001006.2	ENSMUSG00000021313	9.35E−07	Hypertension	[14]
12	51772274	51772705	*Hectd1*	GRCm38:CM001005.2	ENSMUSG00000035247	1.72E−06	Cardiomyopathy	[15]
9	101125023	101153874	*Ppp2r3a*	GRCm38:CM001002.2	ENSMUSG00000043154	3.88E−06	Cardiomyopathy	[16]
1	134834435	134842733	*Ppp1r12b*	GRCm38:CM000994.2	ENSMUSG00000073557	0.00001	Coronary heart disease	[17]
11	4517741	4531305	*Mtmr3*	GRCm38:CM001004.2	ENSMUSG00000034354	0.00001	Coronary heart disease	[18]
17	81428182	81445593	*Slc8a1*	GRCm38:CM001010.2	ENSMUSG00000054640	0.00001	Cardiomyopathy	[19]
X	83878047	83887233	*Dmd*	GRCm38:CM001013.2	ENSMUSG00000045103	0.00002	Cardiomyopathy	[20]
2	76803293	76818816	*Ttn*	GRCm38:CM000995.2	ENSMUSG00000051747	0.00002	Cardiomyopathy	[21]

*Note:* The exact position of each locus is given in terms of chromosome, start and stop position. circRNAs were evaluated by using circTest with false discovery rate of 1%. BH, Benjamini–Hochberg; Ryr2, ryanodine receptor 2; Hectd1, HECT domain-containing E3 ubiquitin protein ligase 1; Ppp2r3a, protein phosphatase 2, regulatory subunit B; Ppp1r12b, protein phosphatase 1, regulatory subunit 12B; MTMR3, myotubularin-related protein 3; Slc8a1, solute carrier family 8 (sodium/calcium exchanger), member 1; Dmd, dystrophin; Ttn, titin.

## Supplementary material

Supplementary Table S1 Complete list of 575 significant circRNAs. Significant circles include a list of 575 significant circRNAs sorted by BH corrected *P* value (sheet 1). Circle raw counts refer to raw counts for detected circRNAs (sheet 2); linear raw counts represent raw counts for linear RNA species (sheet 3); and alternative circRNA counts represent potential alternative splice variants for detected circRNAs (sheet 4).

## Footnotes

The TrackHub containing data of this study in bigWig format is available at https://trackhub.dieterichlab.org/tjakobi/circRNA_heart/hub.txt and a preconfigured instance is accessible via http://tiny.cc/circRNA. Supplementary material associated with this article can be found, in the online version, at http://dx.doi.org/10.1016/j.gpb.2016.02.003.
